# Transcriptomic Analysis of Testicular Gene Expression in Normal and Cryptorchid Horses

**DOI:** 10.3390/ani10010102

**Published:** 2020-01-08

**Authors:** Haoyuan Han, Hong Dong, Qiuming Chen, Yuan Gao, Jun Li, Wantao Li, Ruihua Dang, Chuzhao Lei

**Affiliations:** 1 College of Animal Science and Technology, Henan University of Animal Husbandry and Economy, Zhengzhou 450046, Chinalijun.nn@163.com (J.L.); 2 College of Animal Science and Technology, Northwest A&F University, Yangling 712100, China; 3 College of Animal Science and Technology, Shihezi University, Shihezi 832003, China; 4 Henan Genetic Protection Engineering Research Center for Livestock and Poultry, Zhengzhou 450046, China

**Keywords:** horse, transcriptome, cryptorchidism, testes, differentially expressed genes

## Abstract

**Simple Summary:**

Cryptorchidism is a common congenital malformation that results in impaired fertility in horses. The high abdominal temperature and the effects of this disease lead to differences in gene expression between retained testes and descended testes (DTs). Here, we focus on the genetic effects of cryptorchidism. All the differentially expressed genes (DEGs) between undescended testes (UDTs) and DTs were analyzed in this study. A total of 84 DEGs were associated with functions related to sperm development and male reproductive performance. Our study has provided fundamental transcriptomic data for future studies on equine testes and cryptorchidism.

**Abstract:**

Testes produce sperm, and investigations into gene expression in the testes will enhance the understanding of the roles of testicular genes in male reproduction. Cryptorchidism, the failure of one or both testes to descend into the scrotal sac, is a common congenital malformation in horses. The major clinical consequence of this abnormality is impaired fertility. The aim of this study was to analyze the expression patterns of testicular genes and to identify the differentially expressed genes (DEGs) in testes between cryptorchid and normal horses. In this study, the gene expression patterns in equine testes and the DEGs between mature descended testes (DTs) and undescended testes (UDTs) were identified by RNA-seq and validated by real-time qPCR. Our results provide comprehensive transcriptomic data on equine testes. The transcriptomic analysis revealed 11 affected genes that were downregulated in UDTs, possibly as a result of the higher temperature in the abdomen than in the scrotal sac. These 11 genes have previously been associated with male reproduction, and their downregulation might explain the impaired fertility of cryptorchid horses. Two homozygous missense mutations detected in horses with cryptorchidism were absent in normal horses and were listed as potential pathogenic mutations; these mutations should be verified in the future.

## 1. Introduction

Cryptorchidism, or impaired testicular descent, is the failure of one or both testes to descend into the scrotal sac and is a common congenital malformation in horses. The major clinical consequence of this abnormality is impaired fertility [1] and a significantly increased risk of testicular malignancy due to the negative effects of elevated temperatures on the seminiferous tubules [2,3]. Undescended testes (UDTs) carry the risk of malignant transformation. The interstitial cells of retained testes do not appear to be as sensitive to heat as the cells of the seminiferous epithelium. Thus, while bilateral cryptorchid stallions often exhibit normal secondary sex characteristics, including libido, due to the testosterone (T) produced by interstitial cells, they may not produce viable spermatozoa [1]. Understanding the genetic impact of cryptorchidism on horse testes will provide new insights into the clinical symptoms caused by cryptorchidism. Therefore, we assessed the effects of cryptorchidism on gene expression levels and investigated the genetic factors that lead to impaired stallion fertility.

Testicular descent from the abdomen into the scrotum occurs in two distinct phases: The transabdominal phase and the inguinoscrotal phase [4,5,6]. Failure of this process causes UDTs or cryptorchidism due to impaired development of the gubernaculum [7,8]. Recent studies have revealed that major genetic regulators of testicular descent include the Leydig cell-derived hormones insulin-like factor 3 (*INSL3*) and its receptor relaxin family peptide 2 (*RXFP2*) in humans and mice [7,8,9,10]. *INSL3* is a member of the insulin hormone superfamily expressed in the developing testes [11] and is involved in the transabdominal phase of testicular descent [12]. Mutation of *INSL3* results in bilateral intraabdominal cryptorchidism in humans and mice [7,8]. Furthermore, polymorphisms in the androgen receptor (*AR*), *TGFBR3*, and *Hoxa11* genes and in genetic loci coding for cytoskeleton-associated proteins have been recognized as contributing risk factors for cryptorchidism [13,14,15,16,17]. A chance observation of high incidences of cryptorchidism in humans and animals led to the decision to study impaired testicular descent. Until now, no causal genetic mutation has been found to be the genetic cause for horse cryptorchidism.

To our knowledge, there have been very few studies on the association between cryptorchidism and testicular gene expression levels, and the pathogenesis leading to equine cryptorchidism remains unclear. Herein, we report a complete dataset detailing the testicular transcriptomes of Chinese horses obtained using next-generation sequencing (NGS). NGS technologies, including transcriptome sequencing (RNA-seq), are transcriptomic profiling technologies for mapping and quantifying transcriptomes [18,19,20,21,22,23]. Aberrant transcription and differentially expressed genes (DEGs) between descended testis (DT) tissues and UDT tissues were detected; the findings enhance understanding of the effects of the abdominal environment on gene expression. Furthermore, mutations found in cryptorchid horses but not in normal horses might enable an explanation of equine cryptorchidism in more detail.

## 2. Materials and Methods

### 2.1. Ethics Statement

The protocols involving animals used in this study were approved by the Faculty Animal Policy and Welfare Committee of Northwest A&F University (FAPWC-NWAFU, protocol number NWAFAC1008).

### 2.2. Horse Testicular Tissue Collection

Testicular tissue was obtained from Guanzhong horses (n = 5) and Chakouyi horses (n = 3), of which two individuals (GU4 and CKY2) had unilateral cryptorchidism. Two retained testes and eight normal testes were collected by a professional veterinarian during castration. Guanzhong and Chakouyi are two Chinese horse breeds, the first a crossbred, and the second a native population. The sample information and sampling locations are displayed in Table S1, and the average age of all the samples was 2.0700 ± 1.0593. The testes of GU4 individuals are shown in Figure S1. All the tissue samples were immediately frozen in liquid nitrogen until RNA extraction.

### 2.3. RNA Extraction and Quality Analysis

Total RNA was extracted from the horse testicular tissue using TRIzol Reagent (Invitrogen, Carlsbad, CA, USA) according to the manufacturer’s instructions. RNA degradation and contamination were monitored on 1% agarose gels. The total RNA quantity and purity were checked using a NanoPhotometer^®^ spectrophotometer (Implen, Westlake Village, CA, USA). The RNA concentration was measured using a Qubit^®^ RNA Assay Kit and a Qubit^®^ 2.0 Fluorometer (Life Technologies, Carlsbad, CA, USA). The RNA integrity was assessed using an RNA Nano 6000 Assay Kit and an Agilent Bioanalyzer 2100 system (Agilent Technologies, Santa Clara, CA, USA) with a threshold RNA integrity number (RIN) >7.0.

### 2.4. Library Construction and Sequencing

A total of 1.5 μg of RNA per sample were used as input material for RNA library preparation. Briefly, mRNA was purified from total RNA using poly-T oligo-attached magnetic beads (Invitrogen, Carlsbad, CA, USA). Following purification, the mRNA was fragmented into small pieces using divalent cations at an elevated temperature. Then, the cleaved RNA fragments were reverse transcribed to create the final cDNA library, which was generated using an NEBNext^®^ Ultra^TM^ RNA Library Prep Kit for Illumina^®^ (NEB, Ipswich, MA, USA) according to the manufacturer’s recommendations, and index codes were added to associate the sequences with each sample. To select cDNA fragments of the right length, the library fragments were purified with an AMPure XP system (Beckman Coulter, Beverly, CA, USA). Then, the index-coded samples were clustered on a cBot Cluster Generation System using a HiSeq 4000 PE Cluster Kit (Illumina, San Diego, CA, USA) according to the manufacturer’s instructions. After cluster generation, the prepared libraries were sequenced on an Illumina HiSeq 4000 platform, and 150 bp paired-end reads were generated.

### 2.5. Gene Expression Analysis

The equine genome EquCab2.0 (GCF_000002305.2) and the Y chromosome gene sequences from [24] were utilized as the reference genome and sequences for read mapping using TopHat, and the unique mapped reads were further analyzed. The read count was estimated with htseq-count. The expression levels of the assembled transcripts were normalized, and the DEGs were identified based on their size factors (SFs), relative log expression (RLE), and trimmed means of *M*-values (TMMs) with the DESeq2 R package (1.8.1) (https://bioconductor.org/packages/release/bioc/vignettes/DESeq2/inst/doc/DESeq2.html). The fold changes (in log2 scale) and the corresponding *p*-values and padj values relative to the significance thresholds were estimated for the DEGs according to the normalized gene expression levels. Based on the expression levels, genes with a padj <0.05 and a |log2-fold change| >1 were considered differentially expressed in this study. The functional associations of the DEGs were analyzed, and the protein functions were predicted with the Database for Annotation, Visualization, and Integrated Discovery (DAVID) 6.8 (https://david.ncifcrf.gov/). All searches were conducted with a minimum false discovery rate (FDR) of <0.05 as the threshold.

### 2.6. Real-Time qPCR Verification of the mRNA Sequencing Results

Total RNA was reverse transcribed into cDNA by PrimeScript RT Enzyme in a reaction including gDNA Eraser (RR047, TaKaRa, Dalian, China). Each 10 μL of real-time qPCR included 5 μL of SYBR Green Real-time PCR Master Mix, 0.8 μL of cDNA, and 0.4 μL of each primer. The PCR conditions consisted of 1 cycle at 94 °C for 30 s followed by 40 cycles at 95 °C for 5 s, 61–67 °C for 30 s, and 65 °C for 5 s on a Bio-Rad CFX96 instrument; fluorescence was acquired at 95 °C in the single mode. The relative expression levels were determined using the 2^−ΔΔCt^ method with *GAPDH* as the control.

## 3. Results

### 3.1. Overview of the Sequenced RNAs from Horse Testes

This study used RNA-seq to compare the gene expression of normal testes and retained testes. The RNA-seq generated a total of 73.15 million raw reads, with an average of 7.31 million reads, and a total of 70.80 million clean reads, with an average of 7.08 million (range: 5.98 to 8.88 million) clean reads for each sample (Table 1). The read alignment showed that 87.53% of the reads (61,997,273 reads) mapped to the equine genome (EcuCab2) on average. Of the mapped reads, 84.45% mapped to unique positions, and 1.41% mapped to multiple positions (Table 1). Only the uniquely mapped reads were considered in subsequent analyses.

**Figure 1:**
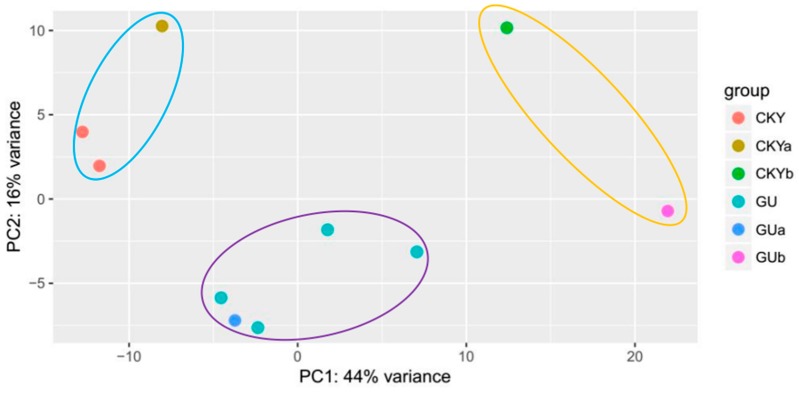
PCA of horse testicular tissues. CKYa represents CKY2a; CKY represents CKY1 and CKY3; GU represents GU1, GU2, GU3, and GU5; GUa represents GU4a; CKY2b represents CKY2b; and GUb represents GU4b.

We conducted a principal component analysis (PCA) on the 10 samples and found that the two UDTs (CKY2b and GU4b), the five normal DTs of Guanzhong horses (GU1, GU2, GU3, GU4a, and GU5), and the three normal DTs of Chakouyi horses (CKY1, CKY2a, and CKY3) tended to form clusters. The testes were classified as normal DTs (PC1 < 10) or UDTs (PC1 > 10) based on PC1 (Figure 1). The current study included three comparisons: A comparison of the gene expression and DEGs between Guanzhong and Chakouyi horses (group 1), a comparison of the gene expression and DEGs between the DTs and UDTs of cryptorchids (group 2), and a comparison of the gene expression and DEGs between the DTs of normal horses and the UDTs of cryptorchid horses (group 3).

### 3.2. Top Genes Expressed in Horse Testicular Tissue

**Figure 2:**
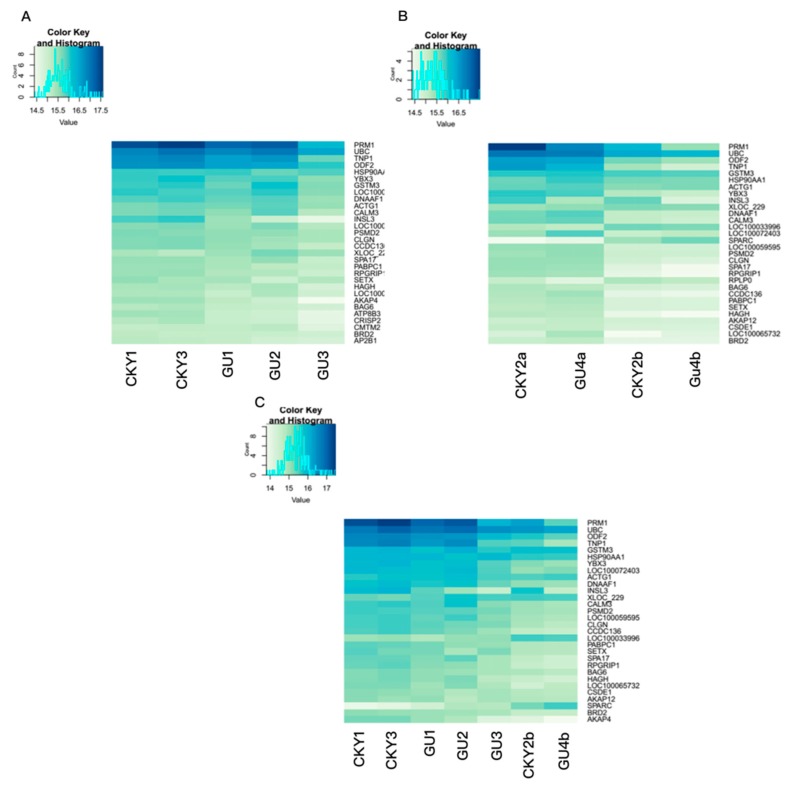
Top 30 genes expressed in horse testicular tissue. (**A**) Gene expression in Guanzhong and Chakouyi horses. (**B**) Gene expression in DTs and UDTs of cryptorchids. (**C**) Gene expression in DTs of normal horses and UDTs of cryptorchids.

The top 30 genes expressed in the testicular tissue in the three comparisons are shown in Figure 2. Strikingly, eight genes, *PRM1*, *UBC*, *TNP1*, *ODF2*, *HSP90AA1*, *YBX3*, *GSTM3*, and *ACTG1*, were included among the top 10 genes in all three comparisons, suggesting that these eight genes are highly expressed in horse testes. As shown in Figure 2B,C, compared to normal DTs, UDTs exhibited downregulation of some genes, including *TNP1*, *DNAAF1*, *CALM3*, *CLGN*, *SPA17*, and *RPGRIP1*; these results reveal the existence of different expression patterns between normal and retained testes.

### 3.3. Differential Gene Expression Analysis

**Figure 3:**
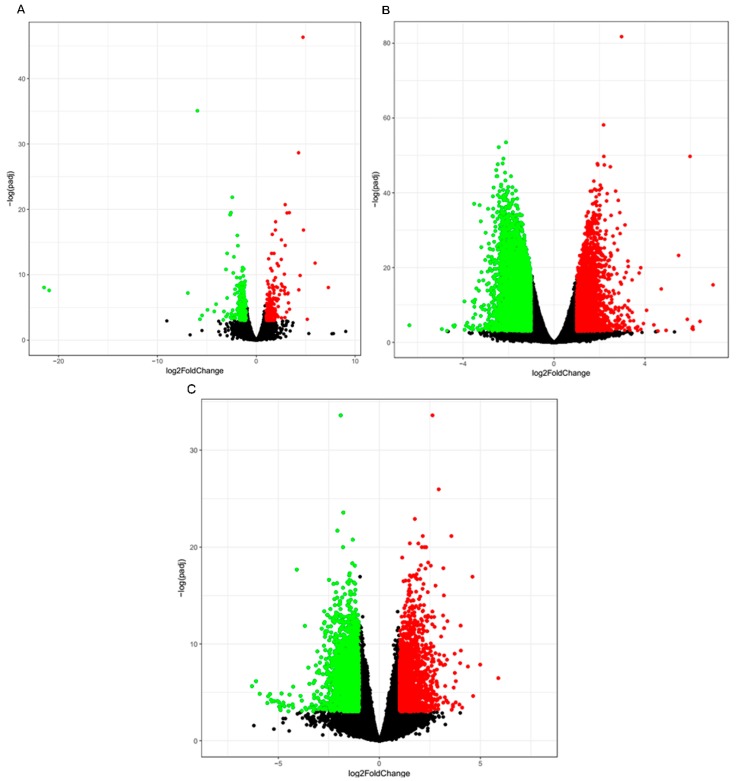
Volcano plot displaying the DEGs in horse testicular tissue. Red dots: upregulated genes. Green dots: downregulated genes. (**A**) DEGs between Guanzhong and Chakouyi horses. (**B**) DEGs between DTs and UDTs of cryptorchids. (**C**) DEGs between DTs of normal horses and UDTs of cryptorchids.

The RNA-seq technique enabled analysis of the differential expression profiles via analysis of the transcript abundance with a high sensitivity. When a *p* < 0.05 and a |log2 fold change| ≥1 were used as cutoffs, a total of 400, 5959, and 5324 DEGs, respectively, were identified in the three comparisons defined above. A total of 191 DEGs were downregulated in the Guanzhong horses compared to the Chakouyi horses while 219 DEGs were upregulated (Figure 3A). Sets of 3341 and 2618 transcripts were downregulated and upregulated in the CKY2b and GU4b testes, respectively, compared to the CKY2a and GU4a testes (Figure 3B). A total of 3049 genes were downregulated in UDTs (CKY2b and GU4b) compared to normal horse testes (CKY1, CKY3, GU1, GU2, and GU3) while 2275 genes were upregulated in these tissues (Figure 3C). Many genes showed differential expression between DTs and UDTs, as shown in Figure 3B,C, indicating that the expression of these genes might be associated with the reproductive performance of cryptorchids. The top 10 genes that were upregulated and downregulated in the three groups are shown in Tables S3–S5.

### 3.4. Real-Time qPCR Validation of the RNA-Seq Results

**Figure 4:**
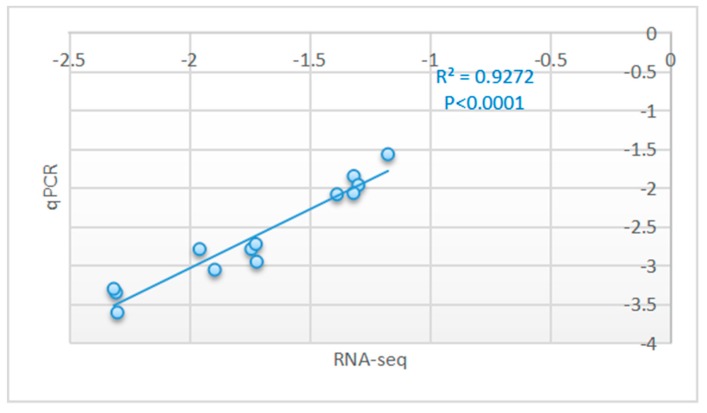
Correlations in the mRNA expression levels of 13 randomly selected DEGs in equine testicular tissues using RNA-seq and qPCR. The x- and y-axes show the log2 ratios of mRNA levels as measured by RNA-seq and qPCR, respectively.

To validate the gene expression results obtained by sequencing, the expression levels of 13 DEGs, namely, *ATP1A4*, *ROPN1*, *NME8*, *CATSPER3*, *CATSPER1*, *AKAP4*, *MNS1*, *CABS1*, *LRGUK*, *TSGA10*, *PRM1*, *CAPZA3*, and *ENKUR*, were analyzed through qPCR. Detailed information on the primers for the 13 genes is given in Table S6. The log2 fold change values determined by RNA-seq and the log2 values determined by qPCR through the 2^−ΔΔCt^ method with normalization to *GAPDH* for the comparisons of these 13 genes are shown in the electronic Supplementary Materials in Table S7. The expression patterns of these 13 genes were consistent with the RNA-seq results according to a Pearson correlation coefficient of 0.9272 and *p* < 0.0001 (Figure 4); quantification of the differential expression levels of the genes indicated significant findings and validated the repeatability and reproducibility of the gene expression data in this study.

### 3.5. Functional Associations of the DEGs

**Figure 5:**
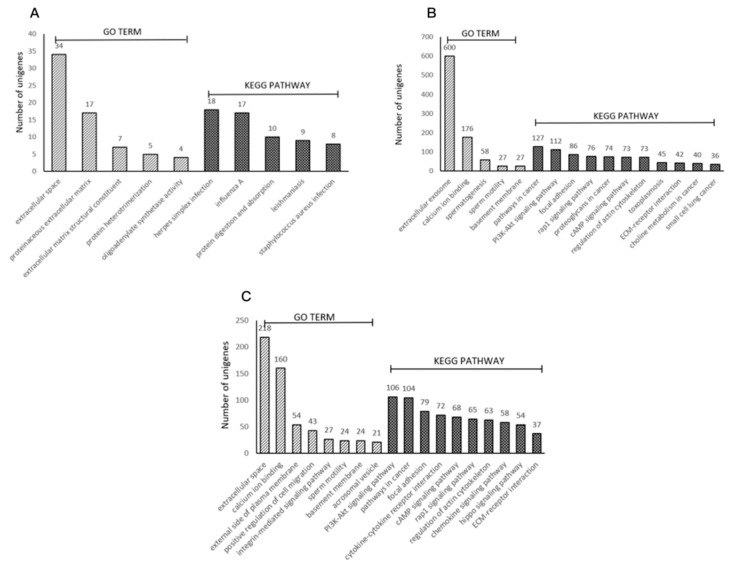
Distributions of the GO and KEGG categories assigned to the DEGs in horse testicular tissues. The x-axis indicates the GO and KEGG groups, and the y-axis indicates the number of genes associated with the group. (**A**) Functional characterization of the DEGs between Guanzhong and Chakouyi horses. (**B**) Functional characterization of the DEGs between DTs and UDTs of cryptorchids. (**C**) Functional characterization of the DEGs between DTs of normal horses and UDTs of cryptorchids. The number of genes is shown on top of each bar.

Various genes cooperate with each other to exercise their biological functions. Accordingly, we performed Kyoto Encyclopedia of Genes and Genomes (KEGG) analysis and Gene Ontology (GO) analysis for terms in the biological process, cellular component, and molecular function categories to further elucidate the biological functions of the DEGs. The DEGs were significantly enriched for 5, 5, and 8 GO terms and 5, 11, and 10 KEGG pathways that met the criterion of an FDR < 0.05 in the three comparison groups (Figure 5). The most significantly enriched GO terms were the extracellular space term (GO: 0005615) with 34 associated genes, the extracellular exosome term (GO: 0070062) with 600 associated genes, and the extracellular space term (GO: 0005615) with 218 associated genes (Figure 5). In particular, the DEGs enriched for the spermatogenesis, sperm motility, and acrosomal vesicle terms are associated with sperm structure and function. The KEGG pathway terms showing the highest levels of significance were the herpes simplex infection term (ecb05168) with 18 enriched DEGs, the pathways in cancer term (ecb05200) with 127 enriched DEGs, and the PI3K-Akt signaling pathway term (ecb04151) with 106 enriched DEGs (Figure 5).

### 3.6. Effects of Cryptorchidism on Gene Expression

**Figure 6:**
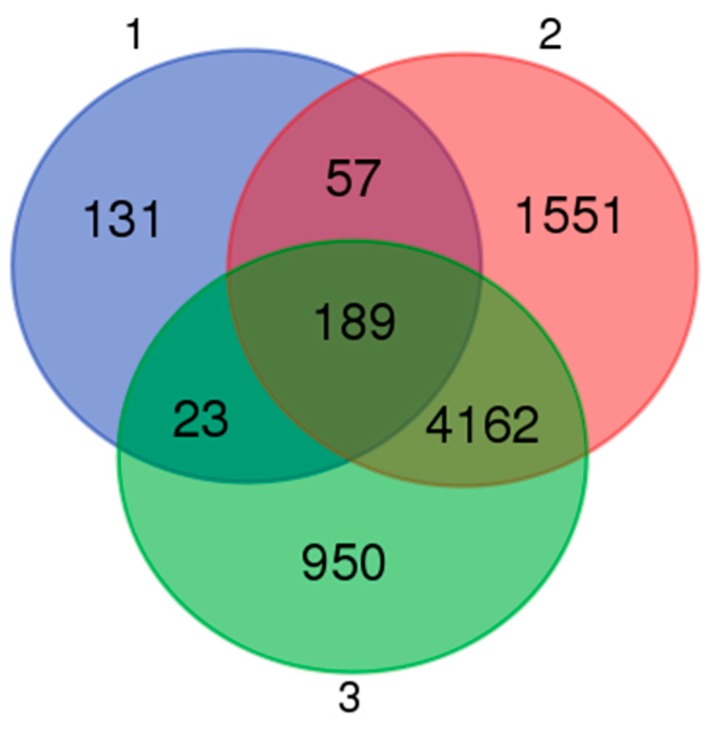
Venn diagram of the unigenes distributed in three groups. Group 1: DEGs between Guanzhong and Chakouyi horses; group 2: DEGs between DTs and UDTs of cryptorchids; group 3: DEGs between DTs of normal horses and UDTs of cryptorchids.

**Figure 7:**
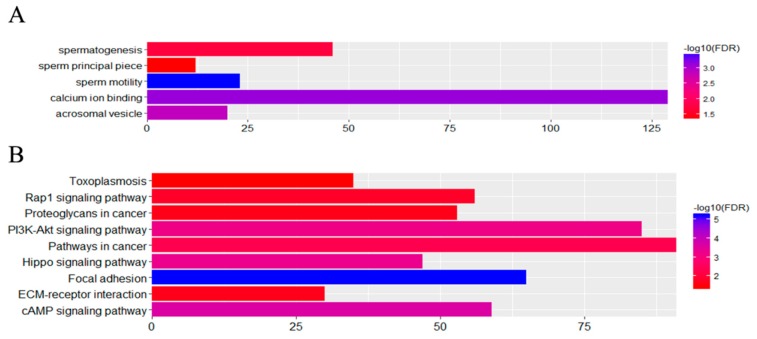
Distribution of the GO and KEGG categories assigned to the DEGs in groups 2 and 3. (**A**) GO terms assigned to 4162 DEGs. (**B**) Eight KEGG pathways assigned to 4162 DEGs.

Our investigations focused on determining the effects of cryptorchidism on testicular gene expression levels in horses. An examination of the overlap of the three groups revealed that 4162 DEGs were shared by groups 2 and 3 when the DEGs between breeds were excluded (Figure 6). These DEGs were assigned to five GO terms and eight KEGG pathways with the criterion of an FDR < 0.05 (Figure 7). It is worth noting that within the GO cellular component category, the acrosomal vesicle (GO: 0001669) and sperm principal piece (GO: 0097228) terms were enriched while within the biological process category, the spermatogenesis (GO: 0007283) and sperm motility (GO: 0030317) terms were enriched (Figure 7). There were 84 DEGs associated with these functions that might influence sperm development and male reproductive performance. Thus, our results support the hypothesis that high abdominal temperature or other factors associated with cryptorchidism impact sperm production and relevant gene expression levels.

### 3.7. Single-Nucleotide Polymorphisms (SNPs) May Cause Cryptorchidism in Horses

Our investigations identified 1,032,196 SNPs in total, of which 146,610 were located in exonic regions. There were 48,083 missense mutations and 77,143 synonymous mutations. Herein, 46 SNPs, including 2 homozygous mutations and 44 heterozygous mutations, were detected in horses with unilateral cryptorchidism but not in normal horses. These variations occurred within 29 genes and are listed in Table 2.

## 4. Discussion

In this study, we compared gene expression levels between UDTs and DTs to determine the genetic effects of cryptorchidism in horses. The statistical power of the analysis of the present research was not so high, because only two samples, belonging to two individuals of Guanzhong and Chakouyi horse breeds, showing unilateral cryptorchidism, were available for the study. This is due to the rarity of the cryptorchidic condition being found in horses. A total of 84 DEGs were associated with functions that might influence sperm development and male reproductive performance; among them, 11 genes were downregulated and were associated with more than two functional terms in our study. These genes have been previously reported to be associated with the fibrous sheaths (FSs) of spermatozoa, nuclear morphology maintenance during meiotic prophase, sperm construction, and male infertility (Table S7). In particular, three of them, CATSPERD, CATSPER1, and CATSPER3, are sperm ion channel proteins involved in sperm hyperactivated motility, which facilitates sperm penetration through the zona pellucida [25,26,27,28]. The expression analysis in the current study, showed that *CatSper1–4* was expressed in horse testes, consistent with previous research [25,29,30]. Furthermore, we found lower expression levels of *CatSper1–4* in UDTs than in normal mature DTs. Other studies carried out on ejaculated sperm have found that *CatSper* transcript levels are positively correlated with sperm motility [31,32]; this association could be the result of essential roles of sperm *CatSper* transcripts in sperm functions, such as motility, capacitation, and the acrosomal reaction [31,33]. Therefore, cryptorchidism leads to reduced expression levels of the *CatSper1–4* genes in UDTs and impaired fertility in horses.

The gene expression levels of *AKAP3* and *AKAP4* were significantly lower in UDTs than in DTs in this study. *AKAP3* and *AKAP4* are the most abundant structural protein components of the sperm FS [34]. It is generally accepted that the FS provides mechanical support and flexibility to the sperm flagellum and provides a scaffold for signaling enzymes and glycolysis that is necessary for hyperactivated motility during capacitation [34,35,36,37]. No research on the relationship between *AKAP* gene expression and cryptorchidism has been reported. Based on this study, *AKAP3* and *AKAP4* are thought to be downregulated in retained testes as a result of the abdominal environment.

*ROPN1* has also been localized to the FS and binds to *AKAP3* and *AKAP4* [34,38]. *ROPN1L* and *ROPN1* compensate for each other in the absence of the opposing protein, possibly to maintain *AKAP3* incorporation in the FS. We found that the *ROPN1* and *ROPN1L* genes both showed significantly lower expression in UDTs than in DTs, indicating that retained testicles have higher frequencies of sperm with dysplasia or do not produce sperm at all. *SPAG6* deficiency causes sperm motility defects and morphological abnormalities [39]. The *SPAG6* gene plays an important role in maintaining flagellar structure and function in mammals [39]. Therefore, cryptorchidism might significantly downregulate the expression of the *ROPN1*, *ROPN1L*, and *SPAG6* genes.

SNP calling was used to clarify the genetic pathogenesis of equine cryptorchidism. A large number of SNPs were screened; however, only 46 missense mutations were detected in horses with unilateral cryptorchidism but not in normal horses. Unfortunately, none of them were consistent with the causal mutations reported previously. Among the 46 missense SNPs, two were homozygous, located within the *PRICKLE3* and *PPP1R42* genes, although no relationship was shown between cryptorchidism and these two genes. The *PRICKLE3* gene has been found to regulate ciliogenesis and cilia growth in gastrocoel roof plates [40]. *PPP1R42* contains a protein phosphatase-1-binding site and translocates from the apical nucleus to the centrosome at the base of the flagellum during spermiogenesis [41]. Therefore, the homozygous SNPs within the *PRICKLE3* and *PPP1R42* genes are considered to be related to horse anorchism, although further verification is needed.

## 5. Conclusions

In conclusion, DEGs between normal DTs and UDTs were identified, and many genes were up- or downregulated in UDTs as a result of cryptorchidism. The decreased expression of 11 genes is considered to be related to the impaired fertility of horses with cryptorchidism. In addition, two homozygous SNPs in the *PRICKLE3* and *PPP1R42* genes are proposed to cause cryptorchidism in horses. A limitation of the present study is the low sample size, since only two individuals showing unilateral cryptorchidism were available for analysis, but this condition is rare in horses. An increased sample size for analysis is aimed for in further research to corroborate the results of the present study. 

## Figures and Tables

**Table 1:** Summary of the transcripts in horse testicular tissues.

Sample	Raw Reads	Clean Reads	Clean Bases	Total Mapped Reads	Multiple Mapped Reads	Uniquely Mapped Reads
CKY1	61,721,592	59,762,158	8,700,950,792	52,179,635 (87.31%)	813,963 (1.36%)	50,385,369 (84.31%)
CKY2a	77,719,686	75,527,312	10,936,704,233	66,175,695 (87.62%)	1,074,061 (1.42%)	63,821,947 (84.50%)
CKY2b	72,480,272	70,588,360	10,234,970,744	62,176,229 (88.08%)	1,002,706 (1.42%)	60,005,651 (85.01%)
CKY3	92,420,914	88,846,736	12,869,802,573	78,285,911 (88.11%)	1,305,326 (1.47%)	75,421,193 (84.89%)
GU1	74,459,300	72,055,254	10,494,742,859	63,316,272 (87.87%)	994,823 (1.38%)	61,130,797 (84.84%)
GU2	73,099,626	70,758,154	10,296,493,984	61,319,858 (86.66%)	1,017,616 (1.44%)	59,085,846 (83.50%)
GU3	79,108,430	76,613,632	11,153,734,663	67,734,267 (88.41%)	1,113,341 (1.45%)	65,306,502 (85.24%)
GU4a	73,056,308	70,657,318	10,241,905,462	60,878,044 (86.16%)	980,165 (1.39%)	58,722,122 (83.11%)
GU4b	65,345,248	63,299,092	9,212,135,314	55,446,209 (87.59%)	869,078 (1.37%)	53,551,478 (84.60%)
GU5	62,041,338	59,931,894	8,714,823,986	52,460,612 (87.53%)	831,832 (1.39%)	50,626,486 (84.47%)
average	73,145,271	70,803,991	10,285,626,461	61,997,273 (87.53%)	1,000,291 (1.41%)	59,805,739 (84.45%)

**Table 2:** Candidate SNPs associated with cryptorchidism.

Gene	NCBI Accession Number	Exon	Nucleotide	Protein	Genotype	
					Normal Horse	Cryptorchid Horse
LOC102147809	XM_005602655.2	exon 1	c.A14G	p.E5G	AA	AG
ZNF37A	XM_005602647.2	exon 5	c.T450A	p.H150Q	TT	TA
DDX60L	XM_014737782.1	exon 22	c.G2747A	p.R916Q	GG	GA
FNIP2	XM_005607793.2	exon 13	c.C2612T	p.T871M	GG	GA
			c.C2231T	p.T744I	GG	GA
C3H16orf46	XM_001501937.3	exon 3	c.G62C	p.S21T	CC	CG
GK2	NM_001256933.1	exon 1	c.A230G	p.E77G	AA	AG
BMP2K	XM_014738530.1	exon 10	c.C1109T	p.T370M	GG	GA
PNPLA8	XM_005609080.2	exon 10	c.C2110T	p.P704S	GG	GA
ALDH9A1	XM_001492799.4	exon 1	c.C125T	p.A42V	CC	CT
LOC100058290	XM_001495326.4	exon 7	c.C479G	p.A160G	GG	GC
GPSM2	XM_001493568.5	exon 14	c.G2006A	p.S669N	CC	CT
TMEM198	XM_001494150.4	exon 1	c.G242T	p.R81L	GG	GT
LOC102148548	XM_014741362.1	exon 3	c.G2650A	p.D884N	CC	CT
			c.G2627C	p.R876T	CC	CG
			c.G2068A	p.V690M	CC	CT
			c.A1679T	p.E560V	TT	TA
			c.A1648G	p.M550V	TT	TC
			c.C581T	p.P194L	GG	GA
			c.A437T	p.Q146L	TT	TA
			c.A369T	p.L123F	TT	TA
			c.T362C	p.M121T	AA	AG
			c.A354C	p.E118D	TT	TG
			c.G278A	p.S93N	CC	CT
PPP1R42	XM_005613086.2	exon 8	c.A799G	p.I267V	AA	GG
TMEM68	NM_001309262.1	exon 1	c.A70G	p.I24V	AA	AG
HACE1	XM_014734298.1	exon 20	c.A2257G	p.M753V	TT	TC
FBF1	XM_005597144.2	exon 9	c.C614T	p.P205L	CC	CT
ABCA9	XM_001916991.3	exon 8	c.T974C	p.V325A	TT	TC
HIRIP3	XM_014730026.1	exon 4	c.A958G	p.K320E	TT	TC
ANKRD31	XM_014730406.1	exon 11	c.G1451T	p.R484L	GG	GT
		exon 15	c.A2804G	p.Y935C	AA	AG
		exon 19	c.T3918A	p.H1306Q	TT	TA
			c.C3922T	p.R1308W	CC	CT
RMDN2	XM_005600050.2	exon 2	c.A13G	p.T5A	TT	TC
VWA8	XM_001493030.5	exon 41	c.G5224A	p.A1742T	GG	GA
CCDC168	XM_014732045.1	exon 3	c.A16781G	p.E5594G	TT	TC
			c.C14732G	p.P4911R	GG	GC
			c.A10753T	p.N3585Y	TT	TA
			c.G10363A	p.E3455K	CC	CT
POM121L2	XM_001495084.5	exon 1	c.A397C	p.T133P	TT	TG
SKIV2L	XM_001492580.3	exon 9	c.C833T	p.P278L	CC	CT
XPO5	XM_005603953.2	exon 19	c.A1996G	p.I666V	TT	TC
MMP9	NM_001111302.1	exon 9	c.A1414G	p.T472A	AA	AG
PSD3	XM_001488201.5	exon 2	c.G144A	p.M48I	CC	CT
PRICKLE3	XM_001495412.5	exon 8	c.G1222T	p.A408S	CC	AA

## Data Availability

The full set of raw data from this study has been deposited in the National Center for Biotechnology Information’s Sequence Read Archive (SRA) and is accessible through the SRA accession number SRR8303651-830365160.

## Supplementary Materials

The following are available online at https://www.mdpi.com/2076-2615/10/1/102/s1. Additional supporting information may be found in the online version of this article. Table S1: Information on the horse testicular samples. Table S2: Top 10 unigenes that were upregulated or downregulated in the testes in Guanzhong horses compared with Chakouyi horses. Table S3: Top 10 unigenes with higher or lower expression in UDTs than in DTs. Table S4: Top 10 unigenes that were upregulated or downregulated in horses with UDTs compared with normal horses with DTs. Table S5: Information on the primers used in qPCR. Table S6: Log2 |fold change| values determined by RNA-seq and log2 values determined by qPCR through the 2^−ΔΔCt^ method for 13 verified genes. Table S7: Functions of 11 genes affected by increased temperature. Figure S1: Testes of GU4 horses. (A): Normal DT; (B): UDT.
