# Supplementary material for: Transcriptomic Analysis of Testicular Gene Expression in Normal and Cryptorchid Horses
Source: Animals (Basel). 2020 Jan 8;10(1):102. doi: 10.3390/ani10010102 (PMC7022935; doi:10.3390/ani10010102)
Supplement: Supplementary file 1 [file animals-10-00102-s001.zip › Supplementary File(s)/Table S1-7.docx]

Table S1 Sample information of horse testes

| Sample name | breed | age | Testis | Location |
| --- | --- | --- | --- | --- |
| GU1 | Guanzhong horse | 2 year-old | Normal | Mei County, Shaanxi Province |
| GU2 | Guanzhong horse | 2 year-old | Normal | Mei County, Shaanxi Province |
| GU3 | Guanzhong horse | 2 year-old | Normal | Mei County, Shaanxi Province |
| GU4a | Guanzhong horse | 2 year-old | Normal | Mei County, Shaanxi Province |
| GU4b | Guanzhong horse | 2 year-old | Retained | Mei County, Shaanxi Province |
| GU5 | Guanzhong horse | 2 year-old | Normal | Mei County, Shaanxi Province |
| CKY1 | Chakouyi horse | 5 year-old | Normal | Tianzhu County，Gansu Province |
| CKY2a | Chakouyi horse | 3 year-old | Normal | Tianzhu County，Gansu Province |
| CKY2b | Chakouyi horse | 3 year-old | Retained | Tianzhu County，Gansu Province |
| CKY3 | Chakouyi horse | 4 year-old | Normal | Tianzhu County，Gansu Province |

Table S2 The top 10 unigenes that were upregulated and downregulated in the testes of Guanzhong horses compared with those in the testes of Chakouyi horses

|  |  | log2FoldChange | pvalue | padj |
| --- | --- | --- | --- | --- |
| up-regulated | LOC100061827 | 5.953863555 | 6.32E-20 | 5.96E-16 |
|  | COL1A1 | 2.422115777 | 6.56E-14 | 3.09E-10 |
|  | FAM198A | 2.561384678 | 1.46E-12 | 3.45E-09 |
|  | FSD2 | 2.636142681 | 2.33E-12 | 4.89E-09 |
|  | COL1A2 | 1.911095195 | 8.46E-11 | 1.23E-07 |
|  | COL6A1 | 1.853950746 | 4.99E-10 | 5.64E-07 |
|  | LOC106783479 | 2.945862256 | 1.83E-09 | 1.89E-06 |
|  | DLK1 | 2.257539702 | 3.03E-09 | 2.72E-06 |
|  | LOC106782461 | 1.379457286 | 2.79E-08 | 1.95E-05 |
|  | ARSE | 3.061282407 | 3.10E-08 | 2.02E-05 |
| down-regulated | ADGRG7 | -4.729512133 | 3.57E-25 | 6.74E-21 |
|  | LOC102149701 | -4.28220639 | 4.95E-17 | 3.12E-13 |
|  | LOC100068487 | -2.926016647 | 2.99E-13 | 1.13E-09 |
|  | LOC100146722 | -3.343492651 | 1.13E-12 | 3.45E-09 |
|  | LOC100630000 | -3.116614548 | 1.42E-12 | 3.45E-09 |
|  | RHBDL3 | -1.962156991 | 8.17E-12 | 1.54E-08 |
|  | FAM25A | -4.780028372 | 3.03E-11 | 5.20E-08 |
|  | GLIPR1L1 | -1.939680533 | 4.13E-11 | 6.50E-08 |
|  | DOCK2 | -1.620852572 | 9.28E-11 | 1.25E-07 |
|  | LOC102147372 | -2.549645077 | 1.98E-10 | 2.49E-07 |

Table S3 The top 10 unigenes that were higher or lower in UDTs than in DTs

|  | genes | log2FoldChange | pvalue | padj |
| --- | --- | --- | --- | --- |
| up-regulated | DLK1 | 2.972096032 | 2.04E-40 | 4.04E-36 |
|  | IL6R | 2.178709054 | 9.24E-30 | 9.14E-26 |
|  | DSC2 | 5.977908214 | 5.28E-26 | 2.09E-22 |
|  | COL1A1 | 2.191718382 | 9.86E-26 | 3.25E-22 |
|  | COL1A2 | 1.909023128 | 1.22E-24 | 2.40E-21 |
|  | ADGRG1 | 2.211854341 | 1.79E-24 | 3.21E-21 |
|  | ROBO1 | 1.926334822 | 2.31E-24 | 3.80E-21 |
|  | PLEKHB1 | 2.475346094 | 2.86E-24 | 4.35E-21 |
|  | MBNL3 | 1.751434592 | 2.90E-22 | 2.87E-19 |
|  | ZNF395 | 2.034707221 | 7.48E-22 | 6.72E-19 |
| down-regulated | FAM71F1 | -2.107528371 | 1.34E-27 | 8.83E-24 |
|  | LOC102147550 | -2.429735264 | 4.91E-27 | 2.42E-23 |
|  | SPATA9 | -2.221102501 | 2.03E-25 | 5.73E-22 |
|  | UBQLNL | -2.251642505 | 7.84E-25 | 1.94E-21 |
|  | IQCF3 | -2.467394829 | 1.12E-24 | 2.40E-21 |
|  | RPH3A | -2.536491772 | 7.07E-24 | 9.99E-21 |
|  | TSSK2 | -2.148378235 | 1.81E-23 | 2.39E-20 |
|  | LOC106782055 | -2.488886205 | 4.55E-23 | 5.41E-20 |
|  | SUN3 | -2.522425995 | 4.66E-23 | 5.41E-20 |
|  | ICA1 | -1.889326472 | 6.10E-23 | 6.70E-20 |

Table S4 The top 10 unigenes that were upregulated and downregulated in horses with UDTs compared with those in normal horses with DTs

|  |  | log2FoldChange | pvalue | padj |
| --- | --- | --- | --- | --- |
| up-regulated | PIWIL4 | 2.629274542 | 2.25E-19 | 4.54E-15 |
|  | EGR4 | 2.936106493 | 8.36E-16 | 5.75E-12 |
|  | MYO1D | 1.756876965 | 3.71E-14 | 1.53E-10 |
|  | PRTFDC1 | 3.562663693 | 2.10E-13 | 6.20E-10 |
|  | EMID1 | 2.146950146 | 2.84E-13 | 7.33E-10 |
|  | HMOX1 | 1.932377067 | 8.28E-13 | 1.71E-09 |
|  | SPON2 | 1.507007726 | 9.19E-13 | 1.72E-09 |
|  | CLEC2L | 2.22849837 | 1.64E-12 | 2.42E-09 |
|  | MID1IP1 | 2.104242378 | 1.57E-12 | 2.42E-09 |
|  | FAM49A | 2.318451506 | 1.97E-12 | 2.71E-09 |
| down-regulated | LOC106781082 | -1.913128001 | 4.41E-19 | 4.54E-15 |
|  | LOC102150060 | -1.781540141 | 1.44E-14 | 7.44E-11 |
|  | ATP1A3 | -2.076910373 | 1.15E-13 | 3.96E-10 |
|  | SPATA16 | -1.317628278 | 6.65E-13 | 1.52E-09 |
|  | ZMYND12 | -1.798258174 | 1.53E-12 | 2.42E-09 |
|  | POU5F2 | -1.334413992 | 1.36E-11 | 1.48E-08 |
|  | XLOC_191 | -4.086906108 | 2.14E-11 | 1.92E-08 |
|  | LOC100068023 | -1.211730902 | 2.29E-11 | 1.96E-08 |
|  | POM121L2 | -1.464527653 | 4.75E-11 | 3.66E-08 |
|  | LOC102149386 | -1.489159654 | 6.28E-11 | 4.47E-08 |

Table S5 Information of primers used in qPCR

|  | full name | Genbank NO. | F | R | length | Tm |
| --- | --- | --- | --- | --- | --- | --- |
| ATP1A4 | ATPase, Na+/K+ transporting, alpha 4 polypeptide | XM_001491321.4 | GTGGGGACCGAATCCCTGCC | ATGCCCCGGGCTGTTCCTTC | 187 | 65 |
| ROPN1 | rhophilin associated tail protein 1 | XM_001501774.3 | TGGCCCAGATGTGGAAGGTGC | GGGGAGGCCCGCTATCATGG | 195 | 67 |
| NME8 | NME/NM23 family member 8 | XM_001492901.4 | AGCTCAGCCCACAAAGGCCA | TGCAAGGTCCACACCAGGCTT | 185 | 67 |
| CATSPER3 | cation channel, sperm associated 3 | XM_005599409.2 | GCCGTGGTATCCGGGTTGAT | CCACCATCAGCTCCCGCTCAA | 192 | 65 |
| CATSPER1 | cation channel, sperm associated 1 | XM_014729546.1 | CCTCGGAAAGCTGGGCCGAA | AGGCTTCGAAGGCCAGGGAC | 186 | 65 |
| AKAP4 | major fibrous sheath A-kinase anchor protein | XM_014728849.1 | AGCCTCGGTGAACAACGCCA | TTGGGAGGCCGCAATCCACT | 178 | 65 |
| MNS1 | meiosis-specific nuclear structural 1 | XM_005603027.2 | CCGGAAACCGCGCAAACACT | CTGCACCATTCGGTCCCTGA | 231 | 63 |
| CABS1 | calcium-binding protein, spermatid-specific 1 | XM_001502161.3 | GGCACTACAAACTCCATGGCT | TTAGCTGGAAGGGCAGGGGT | 245 | 65 |
| LRGUK | leucine-rich repeats and guanylate kinase domain containing | XM_001498102.3 | TCCAGCATTTCGGTCGCCGC | AAGTGCCCCAGCCACGACAT | 170 | 65 |
| TSGA10 | testis specific 10 | XM_014730982.1 | ACGCCCGTCTCCAACTTCCA | CGGGCAATTTCCTCCTGTGCCT | 198 | 65 |
| PRM1 | protamine 1 | NM_001083596.1 | ATGGCCAGATACAGATGCTGCCG | TCGTCTTCTCCTACACCTCAGGACA | 150 | 61 |
| CAPZA3 | capping protein (actin filament) muscle Z-line, alpha 3 | XM_001501974.3 | GGCCACCAGCACTGCCAAAAAT | AGGCGCACAGGACAACATTTCTCA | 220 | 65 |
| ENKUR | enkurin, TRPC channel interacting protein | XM_005606884.2 | CAAAAGCGGCCGGACAGCAG | GCACAGGAGGCTTTTTGGGCT | 226 | 67 |

Table S6 log2 values of fold change by RNA-seq and log2 values of 2^-ΔΔCt^ by qPCR for 13 verified genes

|  | PRM1 | MNS1 | ATP1A4 | CATSPER3 | CATSPER1 | AKAP4 | CABS1 | LRGUR | TSGA10 | CAPZA3 | NME8 | ROPN1 | ENKUR |
| --- | --- | --- | --- | --- | --- | --- | --- | --- | --- | --- | --- | --- | --- |
| RNA-seq | -2.3050 | -1.1756 | -1.3174 | -1.7428 | -1.7207 | -1.9578 | -2.2993 | -1.7245 | -1.2985 | -2.3144 | -1.3186 | -1.8950 | -1.3872 |
| qPCR | -3.3533 | -1.5700 | -1.8517 | -2.7925 | -2.9567 | -2.7942 | -3.6117 | -2.7283 | -1.9667 | -3.3067 | -2.0717 | -3.0600 | -2.0867 |

Table S7 The function of 11 genes affected by increased temperature

| Gene | Full name | Function |
| --- | --- | --- |
| CATSPERD | CatSper channel auxiliary subunit delta | Cation channel sperm associated auxiliary subunit delta. |
| CATSPER1 | Cation channel, sperm associated 1 | The CATSPER proteins play important roles in physiology and fertility of sperm. |
| CATSPER3 | Cation channel, sperm associated 3 | The CATSPER proteins play important roles in physiology and fertility of sperm. |
| ENKUR | Enkurin, TRPC channel interacting protein | The ENKUR protein regulates Ca2+ channels in sperm. |
| ROPN1 | Rhophilin associated tail protein 1 | The protein is in the fibrous sheath of spermatozoa and is involved in sperm motility. |
| NME8 | NME/NM23 family member 8 | The protein is incorporated into the fibrous sheath of mature sperm tails. |
| AKAP4 | Major fibrous sheath A-kinase anchor protein | The protein is the precursor of the major fibrous sheath protein of epididymal sperm. |
| AKAP3 | A-kinase anchoring protein 3 | The AKAP3 protein is the principal piece of spermatozoa and functions as a scaffolding protein in spermatozoa to regulate local cAMP concentrations and modulate sperm functions. |
| SPAG6 | Sperm associated antigen 6 | SPAG6 is a scaffold protein to maintain the structure and activity of sperm flagellum. |
| CCNYL1 | Cyclin Y like 1 | Ccnyll may play an important role in spermatogenesis and causes infertility by affecting sperm morphology and sperm motility. |
| TXNDC8 | Thioredoxin domain containing 8 (spermatozoa) | TXNDC8 is a testis/male germ line specific member of thioredoxin family that accumulates in the superfluous cytoplasm of defective spermatozoa. |
