# Supplementary figures and images for: Transcriptomic Analysis of Testicular Gene Expression in Normal and Cryptorchid Horses
Source: Animals (Basel). 2020 Jan 8;10(1):102. doi: 10.3390/ani10010102 (PMC7022935; doi:10.3390/ani10010102)

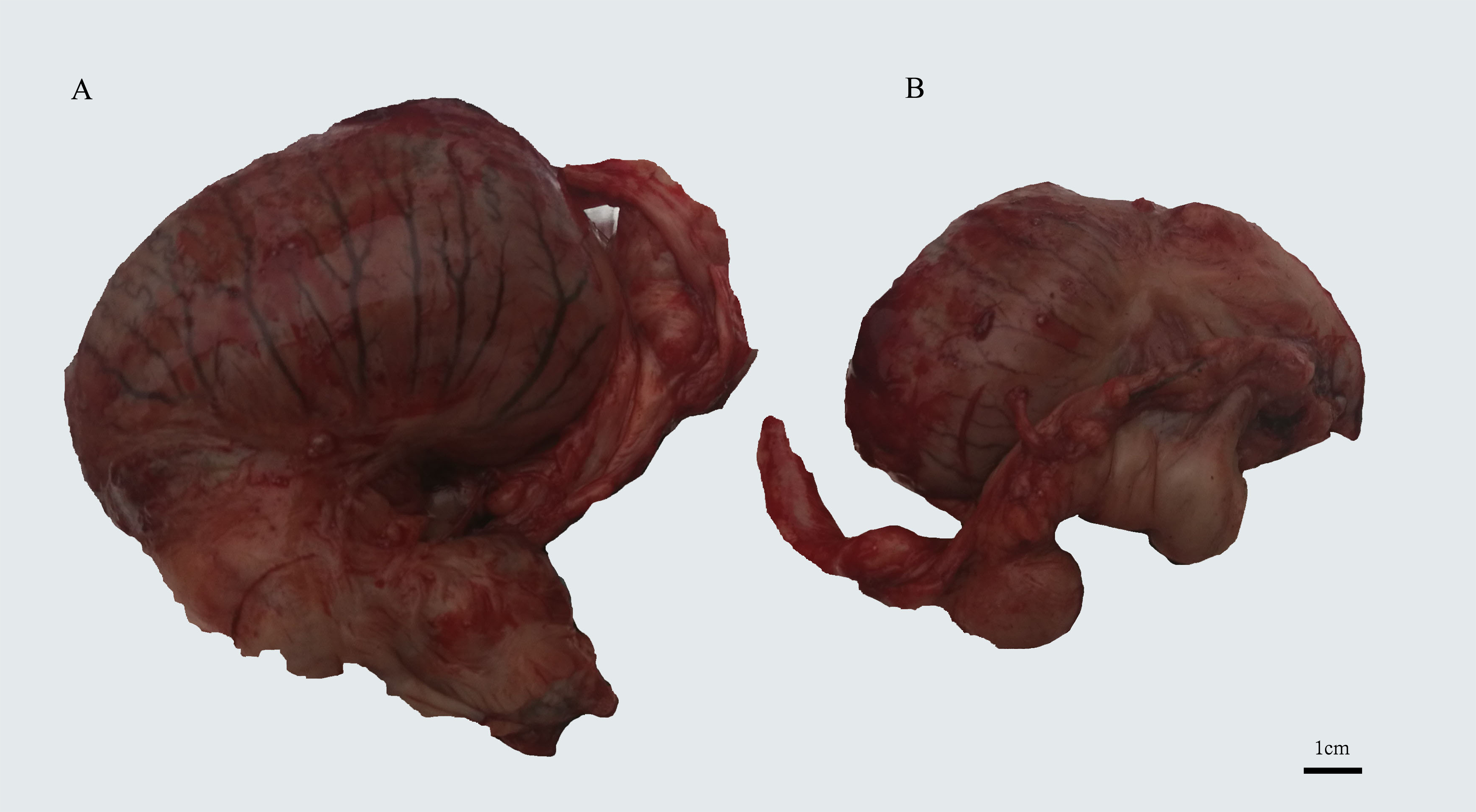

Supplement: Supplementary file 1 [file animals-10-00102-s001.zip › Supplementary File(s)/Figure S1.tif]
